# *Dusukasi*—The Heart That Cries: An Idiom of Mental Distress Among Perinatal Women in Rural Mali

**DOI:** 10.1007/s11013-018-9579-6

**Published:** 2018-04-25

**Authors:** Molly E. Lasater, Madeleine Beebe, Nicole E. Warren, Fatoumata Souko, Mariam Keita, Sarah M. Murray, Judith K. Bass, Pamela J. Surkan, Peter J. Winch

**Affiliations:** 1 Social and Behavioral Interventions Program, Department of International Health, Johns Hopkins Bloomberg School of Public Health, 615 North Wolfe Street, Baltimore, MD 21205-2103 USA; 2 School of Nursing, Johns Hopkins Bloomberg School of Public Health, Baltimore, MD USA; 3 Department of Public Health, University of Sciences, Techniques & Technologies of Bamako, Bamako, Mali; 4 Department of Mental Health, Johns Hopkins Bloomberg School of Public Health, Baltimore, MD USA

**Keywords:** Idioms of distress, Mental health, Mali, Perinatal

## Abstract

Perinatal mental health problems such as depression and anxiety are prevalent in low and middle-income countries. In Mali, the lack of mental health care is compounded by few studies on mental health needs, including in the perinatal period. This paper examines the ways in which perinatal women experience and express mental distress in rural Mali. We describe a process, relying on several different qualitative research methods, to identify understandings of mental distress specific to the Malian context. Participants included perinatal women, maternal health providers, and community health workers in rural southwest Mali. Participants articulated several idioms of distress, including *gèlèya* (difficulties), *tôôrô* (pain, suffering), *hamin* (worries, concerns), and *dusukasi* (crying heart), that occur within a context of poverty, interpersonal conflict, and gender inequality. These idioms of distress were described as sharing many key features and operating on a continuum of severity that could progress over time, both within and across idioms. Our findings highlight the context dependent nature of experiences and expressions of distress among perinatal women in Mali.

## Introduction

Maternal depression is common in low and middle-income countries (LMIC) where risk factors such as poverty, low-educational attainment, and gender-based violence are prevalent (Patel 2007; Rahman et al. 2013). In 2012, the World Health Organization (WHO) published a systematic review of common perinatal mental disorders (CPMDs) among women in LMIC that revealed the prevalence of antenatal and postnatal mental disorders to be 15.5 and 19.8%, respectively (Fisher et al. 2012, 2014). In high-income countries, CPMDs have been associated with impaired child cognitive and emotional development (Kingston et al. 2012; Liu et al. 2017; Murray and Cooper 1997). Growing evidence supports the existence of a similar association in LMICs, where environmental stressors such as poverty place children at high risk for cognitive and psychological delays, and general psychopathology (Goodman et al. 2011; Patel et al. 2003; Wachs et al. 2009; Walker et al. 2007).

The global mental health research community has increasingly come to recognize the impact of cultural context on expressions and experiences of distress, and the importance of accounting for cultural context when designing or modifying diagnostic and treatment systems (Bass et al. 2007; Nichter 2010). Compared to the wealth of epidemiological studies on perinatal mental health in high-income countries, studies of perinatal mental health in LMIC are scarce, particularly ones that use locally-appropriate conceptualization of mental health. This lack of research exists despite the recognized need for appropriate and effective perinatal care in these settings. Accurate epidemiologic and health services research relies on appropriate measurement. When studies fail to account for culture and context in their measurement of mental health problems, they may not only produce misleading or erroneous results, but potentially support the use of scarce resources for non-efficacious interventions and stigmatize already vulnerable populations (Bass et al. 2007; Kohrt and Hruschka 2010). The study of local conceptualizations of mental health can reduce the probability of such harms and facilitate the selection and adaptation of culturally sensitive therapeutic interventions (Hinton and Lewis-Fernández 2010; Kohrt and Hruschka 2010). The study of local syndromes and conceptualizations of mental health, of which idioms of distress are a key component, is a necessary first step in the investigation of mental health, measurement development, and intervention planning (Bass et al. 2007).

The study of idioms of distress, or “social and culturally resonant means of experiencing and expressing distress in local worlds,” allows for the communication of experiential states along a spectrum of mildly stressful to deep suffering that may inhibit functional capabilities (Nichter 2010). Idioms of distress frequently overlap with biomedical diagnostic categories, but may be more useful in the identification of individuals experiencing distress in places where biomedical systems of understanding are not common (Abramowitz 2010; Kohrt et al. 2016). Additionally, idioms of distress may serve as indicators of life distress, including interpersonal problems, personal safety concerns, financial distress, or health-related concerns (Hinton and Lewis-Fernández 2010). Research approaches attentive to idioms of distress and local context are able to produce detailed and nuanced accounts of experiences of suffering and distress that can serve as source material for efforts to sensitize providers to the nature and impacts of mental health problems and contributing factors (Hinton and Lewis-Fernández 2010). Such research approaches furthermore lay the groundwork for culturally and contextually appropriate interventions to improve mental health.

In the West African country of Mali, the mental health system is of very limited capacity. The Ministry of Health lacks an official mental health policy or budget, and there are fewer than 10 psychiatrists in the entire country (WHO 2011). Ranked 175 of 188 countries by the UN Human Development Index, Mali is one of the poorest countries in the world and consistently experiences high rates of maternal and child morbidity and mortality (UNDP 2016). Literacy rates in Mali are particularly low, especially among women, with estimates suggesting that 79% of women aged 15–49 years are illiterate (Cellule de Planification et de Statistique - CPS/SSDSPF/Mali et al. 2013). Polygyny, a form of plural marriage in which a man has more than one wife, is a normative marital system in Mali. In our study region, roughly 44% of women are in polygynous unions (Cellule de Planification et de Statistique - CPS/SSDSPF/Mali et al. 2013). Islam is the majority religion in Mali; however, many Malians also practice animism in tandem with Islam. Animists have a religious worldview characterized by an understanding that spirits exist in natural objects (e.g. plants, animals, and inanimate objects) (Levtzion 1979).

To date, no study has explored the experiences and expressions of perinatal mental health in Mali. To address this gap, we utilized qualitative research methods for the study of perinatal mental health in Sélingué, Mali. This study aimed to describe local idioms of distress and the socio-cultural contexts surrounding perinatal mental health to inform the development of locally-appropriate interventions.

## Methods

We conducted a qualitative study of local conceptualizations of perinatal mental distress in the Sélingué health district of rural Mali during two fieldwork visits conducted over three months: one visit in April and one in June of 2016. Sélingué is comprised of nine health zones (sub-districts), that encompass approximately 91,425 people (Cellule de Planification et de Statistique - CPS/SSDSPF/Mali et al. 2013). Each health zone contains one community health center, which provides antenatal, intrapartum and postpartum care, as well as vaccinations and primary care for all ages. Individuals in Sélingué identify as belonging to the Bambara ethnic group. Given that Sélingué is located on the border with Guinea, there are many influences from the Malinke language on the Bambara dialect spoken in Sélingué, and many people can switch back and forth between the two languages. Compared to Bamako, the capital city of Mali, there is limited ethnic diversity. As Sélingué lacks a dynamic economic sector, few people migrate to the region which is reflected in the overall ethnic and linguistic homogeneity of Sélingué. Life in Sélingué is characterized by profound poverty, as most women live on $1–5 USD/day. Most income generating activities are related to agriculture, fishing and petty trading. Daily life for women in Sélingué is dominated by child rearing, maintaining the family compound, and agricultural work. Some women also participate in economic activities such as selling fire wood and fruits and vegetables at the local market.

Our data collection field team was comprised of the first author and three female Malian research assistants who spoke Bambara, French, and English. The research assistants had at least a bachelor’s degree and work experience in general medicine, qualitative and quantitative public health research, and linguistics. All research assistants participated in a two-day training that covered qualitative research methods, the ethics of human subjects research, and an introduction to perinatal mental health.

The chief medical officer in Sélingué assisted with study recruitment by providing introductions to the directors of each of the five community health centers where we collected data. The directors of each community health center helped the research team identify community health workers (CHWs) and auxiliary midwives to lead study recruitment. CHWs and midwives who were 18 years of age or older and working in Sélingué were also eligible to be sampled as participant informants. With the help of CHWs working in local villages, we approached family compounds to recruit women. Women were eligible if they were 18 years of age or older, pregnant or had a child two years of age or younger, and were currently living in Sélingué. We designed this sampling approach to yield a diverse cross-section of women with regard to mental health and individual circumstances, allowing us to capture a wide range of terms and concepts that were salient and well understood across villages in Sélingué.

Data collection methods included free lists, semi-structured interviews, and focus group discussions (FGDs). All data collection activities were conducted in either Bambara, the lingua franca of southern Mali, or French, the national language in Mali, according to the participants’ preference. We first conducted free lists to identify mental health terminology and problems. Thirty-one individuals, including perinatal women (*n* = 26), auxiliary midwives (*n* = 1), and CHWs (*n* = 4), participated in the free list portion of the study. In the free list interviews, participants were asked “What are all of the problems that affect pregnant women and women who have recently given birth in this community?” Interviewers probed for as many problems as possible. When participants could no longer list terms, they were asked to provide a brief description of each term they had listed. Analysis of free list data took place immediately following data collection. Free lists from all participants were consolidated into one master list. When the research team judged two or more participants to have referred to the same concept with different wording, the team selected the term they felt to be most comprehensive and understood by the population. The final consolidated master free list noted how many participants reported each problem and ranked problems by number reporting in descending order.

Thirty semi-structured interviews were then conducted to gain an in-depth, nuanced understanding of priority mental health problems identified during free listing. Using a purposive sampling strategy, we followed up with perinatal women (*n* = 10), auxiliary midwives (*n* = 1) and CHWs (*n* = 3) from the free listing exercises whom the fieldwork team identified as either being (a) uniquely knowledgeable about mental health and the problems faced by women within their community, or (b) open and able to provide rich, relevant responses. In addition, we recruited CHWs (*n* = 5) and auxiliary midwives (*n* = 6) in Sélingué who had not previously participated in the free lists, and mental health providers working in Bamako (*n* = 4), including psychiatrists, a psychologist and psychiatric medical assistant. Interviews with local women and CHWs focused on developing an in-depth understanding of priority mental health problems, terminology used to describe the problems, perceived causes, and if and how women seek care or manage their distress. Interviews with mental health providers in Bamako elicited descriptions and symptoms of local syndromes or problems identified by freelists in Sélingué, models of causation, pathways to careseeking, and information on informal and traditional mental health treatment modalities found in Mali. Interviews were audio recorded and lasted approximately 60–90 minutes. Detailed notes were taken in French during interviews, as Bambara is primarily a spoken language. At the end of each day, research assistants listened to the audio recording of each interview to elaborate on their notes and transcribe key quotes related to descriptions of idioms of distress. Idioms of distress and key quotes were preserved in Bambara. Interview notes were translated into English and inductively coded using Atlas.ti (Friese 2012). Following coding, we summarized and analyzed the overall themes derived from the inductive coding to draw inferences and characterize our findings.

All study participants provided verbal informed consent in either French or Bambara. The study was approved by the Institutional Review Boards of the Johns Hopkins Bloomberg School of Public Health and the University of Bamako.

## Findings

### *Gèlèya* (Difficulties): Context for Perinatal Mental Distress

**Figure 1:**
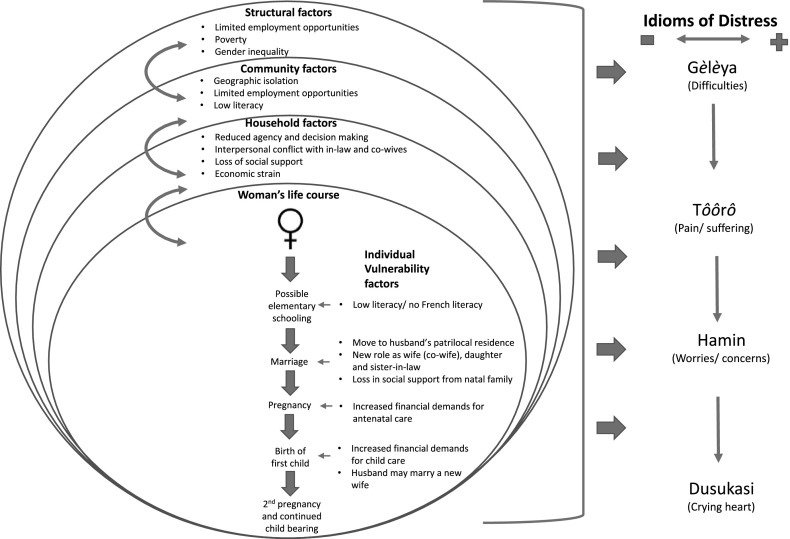
Context for perinatal mental distress

We found that women in Sélingué are born into a life beset with many *gèlèya* (difficulties), including chronic poverty, resource insecurity, and profound gender inequality (see Figure 1). As children, women may have the opportunity to attend primary school for a few years, but most have very limited literacy or are illiterate. Once a woman is married, usually via forced or arranged marriage, she leaves her family compound and moves into her husband’s patrilocal residence with her new parents-in-law, brothers and sisters-in-laws, and possibly co-wives. In Mali, much of the financial burden of providing for the family falls on women, even in cases where men have the financial resources to do so.

Here all of the expenses are women’s responsibility. They should pay for their clothing, clothing of children, and health care costs, buying ingredients for the family sauce, some even buy the cereals which is supposed to be only the role of the man. They do hard work in the field without help of the husband, while all of the benefits that women make from the husband’s field is used for the expenses that the husband should carry. There is only one type of difficulty, the worst kind, and women experience it. [Auxiliary midwife, age 40]

Women described a constant need for financial capital, which they may access through small businesses, such as selling fruits and vegetables in front of their house or at the weekly village market. However, in their new patrilocal residence women expressed having markedly less decision-making power. The majority of decisions pertaining to a woman, including the decision to engage in small income generating activities, are typically the purview of her husband and/or her parents-in-law. While some women’s husbands and in-laws allow them to work, others do not, creating a source of great distress,

I cannot do anything by myself in terms of money, it has to be other people who solve my money problems for me, and if those people refuse or are in lack of money I stay powerless, and this problem stays unresolved. [Woman, age 30]

Given the stressors associated with marriage, an auxiliary midwife in training explained:

I am not yet married, but I have *hamin* (worries) because I see other women married, and their situation makes me scared. There are many men here who want to get married to me but I am concerned a lot. What to do, and then they are all married either to one woman or to two women. [Auxiliary midwife, age 22]

Once married, a woman is expected to bear and raise children, which is associated with additional responsibilities and life stressors. Despite increased financial demands, including user fees for health care, clothes for the child, and medications for a sick child, women are left to their own devices to provide for themselves and their children. One young mother said:

My husband went to Bamako, he works there. He goes back and forth, but the last time he came was when I was 8 months pregnant. Since he has left he hasn’t returned and I gave birth. My baby is 3 months old today. He rarely calls me, he doesn’t send me money, neither for the expenses of the baby or to buy clothes for the baby. He told me to continue with my school, because I should be in 9th grade this year. I asked him ‘how am I going to go to school with a baby, how is the baby going to eat?’ Till now he hasn’t said anything as a response. My father-in-law told me that I cannot go to school while the baby is still young. My mother wanted to take care of my baby so that I can go to school. But what is the baby going to eat? I don’t have money to buy milk for the baby. [Woman, age 18]

Issues related to money between a husband and wife were said to frequently result in marital strain and conflict. As reflected in the prior quote, women described feeling neglected by their husbands and powerless as they are commonly refused access to money-generating activities, and ultimately are left without recourse to address the stressors in their lives. One 31-year old midwife said:

…when you argue with your husband he will tell you to go ask your mom if she receives something (money) from your dad. Your dad will also make you understand that he does it (withholds or controls family finances) too. If he (the father) has the same problem of doing what he wants, then it is not an obligation (for the husband to give money for food and the needs of his children).

Marital conflict also takes place among co-wives. Women expressed how a new wife is frequently favored by the husband, leaving the other wife(s) neglected, both in terms of financial and emotional support. Women also described that while pregnant, husbands may take on girlfriends or additional wives. As a result of these new relationships, a husband may use his money to dote on the new wife or girlfriend while withholding financial and emotional support from his current wife.

The problem is men are neglecting women. Some men when they get married to the second wife, they are finished with the first wife. They wouldn’t have anything in common with the first wife. All of the expenses of the first wife and her children will be in her own responsibility. That causes *dusu tôôrô* (pain of heart). What hurts a lot is when the woman is pregnant, because it is the only moment when the woman can’t work. He didn’t even give money for antenatal care. [Pregnant woman, 21 years old]

### Idioms of Distress

Women described four dominant idioms of suffering and distress (Table 1) that operated on a progressive continuum of severity, both across and within idioms. As detailed above, *gèlèya* (difficulties), and the low levels of distress that it produces, initiates the sequence. Over time, this can lead to *tôôrô* (pain), and too much *tôôrô* (pain) can then result in *hamin* (worry or concern). Continued experiences with pain and worry can eventually culminate in *dusukasi* (crying heart).

**Table 1:** Bambara idioms for perinatal mental distress

Bambara idiom	English translation
*Dusukun chauffé* ^a^	Hot heart
*Dusu tôôrô*	Pain in the heart
*Dusukasi*	Crying heart
*Fari faga*	Weak body, fatigue, low energy
*Fatoya*	Crazy, psychotic
*Gèlèya*	Difficulties
*Hamin*	Worry, concerns
*Kato I kelena*	To be alone
*Kelena kuma*	Talking to oneself
*Tôôrô*	Pain, suffering

^a^First word is Bambara, second word is French

### Tôôrô

In its routine use, the local term for pain, *tôôrô*, can denote a wide range of suffering and misfortune, both health and non-health related. As a health condition, participants described *tôôrô* as an internal condition that can affect the body, mind, or both. Cuts or bruises, bodily pain, and fatigue were described as examples of physical or somatic *tôôrô*, while experiences of *gèlèya,* worry, rumination, or a pained heart or soul invoked mental *tôôrô*.

Explanations of *tôôrô* signaled that it can represent both an emotional reaction to a stressor, as well as symptoms of *hamin* (worry) or *dusukasi* (a crying heart) (see discussion below). To learn more about what people mean when they report *tôôrô*, we spoke with mental health professionals at the University of Bamako:

It will provoke insomnia, and it is a cause of social isolation; the person feels isolated, humiliated. It is a cause that explains the behavioral problems, the fact that the person is and feels that they are in this situation, which causes, effectively, the symptoms. It is not a real sickness, but it is a factor that leads to sickness. [Psychologist, male]

Another professional spoke to the meaning of *tôôrô* in its widest sense,

In my opinion *tôôrô* is equal to stress, mentally. It’s our way to express the pain in us, let it be mental or somatic… Women may have a problem in the family or in the work… Generally they express themselves as ‘I am pained, I am worried, I am concerned.’ [Psychiatry resident, female]

Later this psychiatry resident remarked that patients suffering from *tôôrô* often experience somatic symptoms (specifically stomach or chest pain) that can prompt them to seek care from general practitioners.

Participants reported that in chronic experiences of *tôôrô,* increasing severity can result in diminished functionality and motivation. One mother described functional impairment across life domains:

I didn’t like to get dressed. I diminished taking care of my husband. I couldn’t eat well, even if I wanted to eat, once I started to think about the problem, I didn’t want to eat anymore. I didn’t take care of myself. [perinatal woman, age 28]

### Hamin

Most participants reiterated that *hamin* (worry) is experienced following *gèlèya* (difficulties) and *tôôrô* (pain). *Hamin* translates to worry or concerns. A psychologist provided the following explanation of people suffering from *hamin*: “They have lost their energy, this joy of life is gone, when you have *hamin* you are not calm in your mind… and little things can bother you” [Psychologist, male]. *Hamin* communicates an internal condition characterized by intense thinking and rumination *(miiri miiri)*, withdrawal and social isolation, being silent, and talking to oneself. *Hamin,* also invokes an embodied experience of a “hot head” or “a mind that won’t sit still.” Participants mentioned several behavioral indicators for *hamin,* such as walking back and forth aimlessly, having a serious face, nonsense talk, talking too much or not talking at all, and either not greeting people or doing a very short greeting, which is widely seen as a violation of social norms in Malian culture.

*Hamin* is situated in the center of the continuum that goes from “small” *hamin* (worries and concerns) to “big” *hamin* (intense rumination and anxiety). One mother described an example of small *hamin* as the worries related to finding enough money for the ingredients for a meal when it was her turn to cook for the family, which may cause sleep disturbances and changes in appetite. While this kind of small *hamin* might resolve on its own (for instance after a woman’s turn cooking had passed), *hamin* in general was described as a pervasive issue that weighs on women. Multiple and chronic experiences of *hamin* was described as leading to big *hamin,* which, as described by one participant, could ultimately have serious mental and social consequences.

The person (who experiences big *hamin)* loses weight and looks bad. The person becomes like *fato* (crazy). The person talks alone to herself because she has a lot of *hamin*. The person sits next to people without being present and is silent. The person is always *miiri miiri* (thinking). Even if the person hangs out in a group, you will find out that she is not thinking of what the group is saying or talking, but she is thinking all of her problems in the family, she is not there. [CHW, age 19]

A metaphor used to describe those suffering from big *hamin* was “being stuck between two rocks with nowhere to go,” alluding to women having no options to resolve their *hamin* once it has reached this level of severity.

Additionally, in describing emotional responses to *hamin,* participants frequently mentioned *kashi* (crying):

Every time you see her she will be thinking, even if she came to hang out she will just think and cry. During the social gathering she will be thinking, she will not take part in the conversation, and if she wants to talk about the problem she will just cry, and people will pity you (Pregnant woman, age 19).

### Dusukasi

Participants widely reported that *dusukasi* (a crying heart) is the peak of mental distress. Similar to *tôôrô* and *hamin*, *dusukasi* was also described as being experienced on a continuum. Our participants conveyed the notion of ‘feeling’ *dusukasi* as a symptom, whereby one can experience a normative response of feeling sad in reaction to some situation or experience, but without ‘having’ *dusukasi.* At the opposite end of the continuum is ‘having’ *dusukasi*, which is considered serious and framed by idioms of distress related to fatigue, rumination, social isolation, difficulty with concentration, and sadness.

Your body is weak, your head hurts, you think over and over the same thing, you are physically there but your mind is elsewhere. When your body is *fari faga* (weak), talking to other people disturbs you, you prefer to be *kato I kelena* (alone), you don’t want to do anything, you can’t even work or talk to people, it doesn’t please you. You become silent, have lots of worries, which makes you have *dusukun chauff*é (a hot heart). All of this pains your soul, and you stay alone like that. [CHW, female, 20 years]

Given the duality of *dusukasi,* participants stressed its prevalent nature in their communities, and the potential for ‘feeling’ *dusukasi* to evolve into ‘having’ *dusukasi*: “*Dusukasi* risks to kill all of the women here, they are just barely living. We always say that these women are traumatized, but it is being worried and feeling *dusukasi* which is the basis of [having] *dusukasi.”* (Pregnant woman, age 18)

Participants described how ‘having’ *dusukasi* may lead women to internalize the social and marital conflicts in their lives, causing her to “not like herself”, “neglect herself” or feel “demolished”. Women with extreme cases of *dusukasi* may become *fato* (crazy), because they become detached from reality, silent, and withdrawn. In addition, participants reported that *dusukasi* can result in death or suicide if adversities are not resolved: “They (women) say to their children ‘I am going to go. When you grow up they will tell you where I am. That is better than if we show you my grave.’” [Pregnant woman, age 18]

Given the progressive nature of the idioms of distress in terms of severity, both across and within idioms, many participant descriptions of *tôôrô* and *hamin* suggested a large degree of overlap or shared features with *dusukasi.* Regarding *tôôrô*, participants indicated an interrelation between mental and physical *tôôrô* that resembled idioms of the heart related to *dusukasi*:

For me, all of *tôôrô* is about the heart. If your heart is *tôôrô*, your body gets *tôôrô*. If you heart is *tôôrô* your body becomes dead, you can’t do anything, sometimes you don’t sleep. Like if you are in a large family and you are having disputes all the time with people, all this can bring you *tôôrô*, so if the heart is *tôôrô*, the body will be *tôôrô* too. (Auxiliary midwife, female)

## Discussion

Among perinatal women in rural Mali, idioms of distress communicate individual experiences of suffering; however, these idioms are interpersonal and social in nature, deeply rooted within a context of poverty and gender inequality. Women used idioms of distress to communicate difficulties (*gèlèya)*, stress, pain, and suffering (*tôôrô*), worries (*hamin*), and a crying heart (*dusukasi*), which operate progressively along a continuum of severity of distress that increases with time, both within and between idioms. For example, *hamin* was frequently described as an emotional response to a concerning exchange or situation, that can be experienced as a one-time event, a local syndrome, or a symptom of *dusukasi*. Whether *hamin*, or the condition of interest, is experienced as a symptom or syndrome is dependent on the duration of *hamin*. With the passage of time, or reoccurrence of distressing experiences, one’s *hamin* can worsen if not addressed or resolved, and lead to *dusukasi.* A study among traditional healers in The Gambia described a similar temporal relationship between trauma-induced disorders and affective disturbances, whereby an individual may develop an anxiety-like condition following a traumatic event, and as a result of rumination develop a more serious ‘thinking sickness’ characterized by sleep and appetite disturbances, sadness, apathy, and social isolation (Fox 2003). Similarly, Hinton and Lewis observe this facet of idioms of distress, noting that when an idiom of distress is experienced as a near continual condition, the idiom is frequently perceived to predispose to other idioms of distress that may be experienced both chronically or episodically (Hinton and Lewis-Fernández 2010).

As has been observed in other studies of perinatal mental health in low-income countries, the local cultural and socio-economic context was seen by women in rural Mali as exerting great influence on the mental health of pregnant women and young mothers (Avotri and Walters 2001; Fisher et al. 2012). As women move through seminal life phases they amass increasing levels of responsibility, adversity, and stress. For women in rural Mali, the stressors and adversity associated with living in chronic poverty are greatly compounded after marriage, a pivotal and transformative life event. With marriage, individual responsibilities and stress sharply increase, while at the same time, women’s resources for coping are limited by the decreases in social support and personal agency that occur as they relocate to their husband’s patrilocal residence.

Studies from West Africa describe how marriage constitutes a social relationship between two kin groups, but not one in which the sharing of resources or decision making by a husband and wife are common (Avotri and Walters 2001; Manuh 1994; Potash 1995). Rather, marriage acts as a means to transfer control and influence over women’s individual rights, including rights to labor and reproduction, from the natal to the marital household (Adams and Castle 1994). Women described demanding workloads, limited control over their work, and generally an inability to earn income, after being married as factors contributing to distress. Despite widely held social norms dictating that husbands are responsible for providing grains for the family and funds for expenditures associated with children’s schooling, medical visits, and clothing, husbands were frequently said to refuse to pay for such expenses. As such, women are forced to bear the majority of financial responsibilities and associated heavy workloads, while gender roles and norms simultaneously limit women’s agency, decision-making power, and access to resources. While having a trusting relationship, particularly with an intimate partner, are commonly found to be protective of mental disorders or distress, such relationships appear to be rare in rural Mali (Fisher et al. 2007; O’Hara and Swain 1996). Instead, traditional marital systems coupled with gender and power inequality may constitute vulnerabilities for perinatal mental distress.

Our findings are consistent with other studies demonstrating the role of household dynamics, interpersonal conflict, and related factors in the production of perinatal mental distress (Abiodun 2006; Chandran et al. 2002; Fatoye et al. 2004; Fisher et al. 2007; Shepard 2013). Study participants described interpersonal conflicts with their husbands, co-wives, and parents-in-law. Conflicts with husbands, as described above, were typically centered around the negotiation of household finances and amount of time spent with each co-wife, issues that also generated conflict among co-wives themselves. Studies in Nigeria and Nepal have documented increased symptom scores for CPMDs among women in polygynous marriages compared to monogamous marriages, citing marital discord and friction and low spousal support as mechanisms linking polygyny to depression (Dørheim Ho-Yen et al. 2007; Fatoye et al. 2004). Among our study population, polygyny serves as an example of the “co-operative conflict” model, whereby co-wives must cooperate in their household duties while, at the same time, competing for the attention, parental investment, and material and financial resources of the husband (Bove and Valeggia 2009; Sen 1990). In rural Mali, the mental health impacts of polygyny may be further compounded by the social subordination of women to their husbands and husband’s family, including older female in-laws and co-wives, who may have greater access to resources and social capital. Women also described difficult interpersonal relationships with in-laws in relation to a lack of autonomy regarding personal decision making. Similar to polygyny, this may reflect the hierarchical nature of household relationships and the authority of husbands and in-laws over women’s lives.

*Idioms* of distress are highly dependent on culture and context, reflecting the complexity of their uses and dynamic interactions with other idioms of distress. While idioms of distress are characterized by distinctive elements, the boundaries between idioms are fluid with many shared features, reflecting their progressive nature. Revisiting the example of *hamin* from above, depending on the duration or chronicity of distressing experiences and situational factors, *hamin* may denote different meanings, such as a symptom of *dusukasi* or a local syndrome of anxiety. Similarly, a study among the Highland Quechua in Peru describes idioms of distress as being polysemic and multi-vocal in nature, as they are entities dependent on risk exposures, individual contexts, as well as larger social contexts spanning colonial influences, poverty, and globalization (Pedersen et al. 2010). As such, it is important to caution the labeling of these idioms, particularly in comparison to Western diagnostic constructs, given that the meanings and interpretations of idioms of distress vary according to individual and experience.

Future studies on idioms of distress must examine the nuanced local and contextualized meanings of idioms of distress in order to understand how suffering is understood and presented in a given cultural context. Evidence from research that used locally validated screeners indicate that even when a cultural syndrome is non-specific to biomedical diagnostic criteria, the use of local idioms of distress in interventions can facilitate interpersonal communication, accurate identification of elevated levels of distress, and therapeutic rapport and efficacy (Abramowitz 2010; Hinton and Lewis-Fernández 2010; Kohrt and Harper 2008; Kohrt et al. 2016). Additionally, in settings such as rural Mali where perinatal mental health problems are rooted in social and economic strain, mental health interventions necessitate multidisciplinary approaches that focus on investments in women’s empowerment as well as individual and community strengthening.

Several limitations must be noted while interpreting findings from this study. While our study produced rich and nuanced data on maternal mental health, we only worked one region of Mali, among women of a single ethnic and linguistic group. Therefore, the results of our study may not necessarily reflect other regions of Mali. Given the rich ethnic, linguistic, and socio-economic diversity in Mali, any single model of idioms of distress related to maternal mental health in Mali would likely be an oversimplification. Another limitation of this study is that the first author was not fluent in Bambara or French. As such, when data collection activities were conducted in Bambara, the data went through three rounds of translation from Bambara, to French, to English. Through this process of translation, meanings ascribed to the data may have changed. To overcome this challenge, we worked with research assistants who were fluent in French and Bambara, and one who was also fluent in English, and all data was translated from French to English by a single research assistant. Additionally, the team held debriefings at the end of each day of data collection to review the interviews that had been conducted and to develop group consensus on how to interpret the meaning of the translated data.

In summary, idioms of distress among perinatal women in Sélingué, Mali reflect and communicate substantial challenges and hardships in life that create vulnerabilities and opportunities for mental distress. A complex web of interacting factors related to poverty and gender inequality lies at the root of maternal mental distress, and one form of distress can interact circuitously to further perpetuate vulnerabilities for other forms of distress. Understanding how people label and confer meaning to life’s stressors through the study of idioms of distress is the first step in developing locally adapted and validated screeners to measure mental distress, and ultimately to developing locally relevant mental health interventions.
